# Blockage of KHSRP-NLRP3 by MCC950 Can Reverse the Effect of Manganese-Induced Neuroinflammation in N2a Cells and Rat Brain

**DOI:** 10.3390/ijms232113224

**Published:** 2022-10-30

**Authors:** Sharad Singh, Ibrahim Ahmed Shaikh, Sunil S. More, Mater H. Mahnashi, Hailah M. Almohaimeed, Mohamed El-Sherbiny, Mohammed M. Ghoneim, Ahmad Umar, Harshit Kumar Soni, Himanshu Agrawal, Basheer Ahmed Mannasaheb, Aejaz Abdullatif Khan, Uday M. Muddapur, S. M. Shakeel Iqubal

**Affiliations:** 1 School of Basic and Applied Sciences, Dayananda Sagar University, Bangalore 560111, Karnataka, India; 2 Department of Pharmacology, College of Pharmacy, Najran University, P.O. Box 1988, Najran 66462, Saudi Arabia; 3 Department of Pharmaceutical Chemistry, College of Pharmacy, Najran University, P.O. Box 1988, Najran 66462, Saudi Arabia; 4 Department of Basic Sciences, College of Medicine, Princess Nourah bint Abdulrahman University, P.O. Box 84428, Riyadh 11671, Saudi Arabia; 5 Department of Basic Medical Sciences, College of Medicine, AlMaarefa University, P.O. Box 71666, Riyadh 11597, Saudi Arabia; 6 Department of Anatomy, Faculty of Medicine, Mansoura University, Mansoura 35516, Egypt; 7 Department of Pharmacognosy and Medicinal Plants, Faculty of Pharmacy, Al-Azhar University, Cairo 11884, Egypt; 8 Department of Pharmacy Practice, College of Pharmacy, AlMaarefa University, Dariyah, P.O. Box 71666, Riyadh 13713, Saudi Arabia; 9 Department of Chemistry, Faculty of Science and Arts, Najran University, P.O. Box 1988, Najran 11001, Saudi Arabia; 10 Promising Centre for Sensors and Electronic Devices (PCSED), Najran University, P.O. Box 1988, Najran 11001, Saudi Arabia; 11 Department of Zoology, Government Science College, Pandhurna 480334, Madhya Pradesh, India; 12 Jubilant Biosys Limited (Discovery Biology), Bangalore 560022, Karnataka, India; 13 Department of General Science, Ibn Sina National College for Medical Studies, P.O. Box 31906, Jeddah 21418, Saudi Arabia; 14 Department of Biotechnology, KLE Technological University, BVB Campus, Hubballi 580031, Karnataka, India

**Keywords:** neuroinflammation, KHSRP, manganese neurotoxicity, N2a cells, Parkinson’s

## Abstract

Manganese neurotoxicity has been reported to cause a neurodegenerative disease known as parkinsonism. Previous reports have shown that the expression of the KH-type splicing regulatory protein (KHSRP), a nucleic acid-binding protein, and NLRP3 is increased upon Mn exposure. However, the relation between these two during Mn toxicity has not been fully deduced. The mouse neuroblastoma (N2a) and SD rats are treated with LPS and MnCl_2_ to evaluate the expression of KHSRP and NLRP3. Further, the effect of the NLRP3 inhibitor MCC950 is checked on the expression of NLRP3, KHSRP and pro-inflammatory markers (TNFα, IL-18 and IL-1β) as well as the caspase-1 enzyme. Our results demonstrated an increment in NLRP3 and KHSRP expression post-MnCl_2_ exposure in N2a cells and rat brain, while on the other hand with LPS exposure only NLRP3 expression levels were elevated and KHSRP was found to be unaffected. An increased expression of KHSRP, NLRP3, pro-inflammatory markers and the caspase-1 enzyme was observed to be inhibited with MCC950 treatment in MnCl_2_-exposed cells and rats. Manganese exposure induces NLRP3 and KHSRP expression to induce neuroinflammation, suggesting a correlation between both which functions in toxicity-related pathways. Furthermore, MCC950 treatment reversed the role of KHSRP from anti-inflammatory to pro-inflammatory.

## 1. Introduction

Manganese is one of the most abundant (12th most abundant) elements on Earth [1]. It is crucial for the proper functioning of various enzymes involved in metabolism as well as blood clotting, protein glycosylation, immune function and reproduction [2,3]. Elevated levels of Mn in the brain usually occur through overexposure via environmental sources, occupational exposures or dietary exposures. Overexposure to Mn is reported to occur throughout the central nervous system and affects the normal functioning of both motor functions and higher-order cognitive functions. Disease conditions related to intoxication with Mn are found to be harmful and could cause manganism [4]. Manganism is also called parkinsonism, because its symptoms are similar to those of Parkinson’s disease [5]. Manganese toxicity is thought to be mediated by oxidative stress, neuroinflammation, mitochondrial dysfunction, protein misfolding and apoptosis [3,5,6]. Manganese can pass the blood–brain barrier in a DMT-1-mediated and/or transferrin-mediated pathway. Thus, it could deposit onto the brain striatum and globus pallidus, which could result in neuroinflammation and neurodegeneration [7,8,9]. Despite the work undertaken in this area, the detailed mechanism of manganese toxicity is poorly understood.

In the case of neurodegenerative disease, microglia cells are the population of macrophages that play a special role in clearing damaged neurons from the CNS and are considered immune sentinels that are competent in initiating a dominant inflammatory response [10]. The immune responses in the CNS are associated with pathogen-associated motifs from an extracellular source; these pattern recognition receptors include Toll-like receptors and Nod-like receptors [11].

Once the pathogen or infectious agent is recognized by an immune system within the CNS, a cascade of activation pathways is triggered, releasing cytokines and chemokines. Manganese has been reported to induce immune activation, releasing pro-inflammatory cytokines such as IL1β [12]. Although the mechanism of Mn-induced immune activation/response is poorly understood, IL1β is the mediator of the inflammatory cascade; it is produced as an inactive precursor (36 kDa, called pro-IL1β), and on encountering the pro-inflammatory stimuli pro-IL-1β is cleaved into the biological active form (17 kDa, called mature IL1β) [13,14]. The regulation and the maturation of IL1β represented by inflammasome involves NLRP3 (NLR family, pyrin domain containing 3)-CASP1 (caspase 1). The NLRP3-CASP-1 inflammasome pathway has been reported as a multifaceted innate immune receptor because of its multiple roles in inflammation, and can be activated by a broad range of stimuli. No matter how but Mn, a naturally found heavy metal, activates the innate immune response through the NLRP3-CASP1 inflammasome pathway in a manner as yet to be explored [15].

The KH-type splicing regulatory protein (KHSRP) is an AU-rich RNA-binding protein (ARE-BP) [16]. KHSRP negatively regulates a subset of cytokines and chemokines by modulating translational silencing, RNA instability, micro-RNA maturation and transcriptional repression. It has been reported that KHSRP could play a role in manganese-induced neurotoxicity [17]. KHSRP post-transcriptionally regulates the expression of iNOS. This regulation affects the p38 MAPK and NF-κB signaling pathways [18,19,20,21]. KHSRP has also been reported to promote the destabilization of β-catenin and to thereby regulate the Wnt signaling pathway [22,23,24,25]. These findings indicate that KHSRP might play a deeper role in inflammation and apoptosis than thought. Shi et al. [17,26] reported the upregulation of KHSRP in rat striata in PC-12 cells upon Mn exposure. They also showed that increased KHSRP expression was correlated with the upregulation of p53, Bax and caspase-3. These reports imply that KHSRP might play a role in Mn-induced neurotoxicity; however, the detailed mechanism of the role of KHSRP in Mn-induced neuroinflammation is not known.

In this study, we investigated the role of KHSRP in Mn-induced neurotoxicity and inflammation. We also investigated the possible signaling mechanism mediating the role of KHSRP in Mn-induced neuroinflammatory response. Our findings provide more insight into the role KHSRP plays in neuroinflammation in general and specifically in Mn-induced neuroinflammation.

## 2. Results and Discussion

### 2.1. ManganeseCl_2_ Induces Toxicity in N2a Cell Lines

Manganese is a key environmental pollutant. Exposure to manganese (Mn) could lead to neurodegenerative disorders such as Parkinson’s disease (PD). Manganese toxicity could affect the nigrostriatal neuronal circuitry of the central nervous system, which leads to behavioral and motor impairments. However, the mechanism of this toxicity is not well understood. To decipher the signaling of Mn toxicity and the roles of KHSRP and NLRP3 in Mn-mediated toxicity, the mouse neuroblastoma cell line N2a was treated with various concentrations of MnCl_2_ for various time points and its effect on N2a cell viability, KSHRP expression and secretion of pro-inflammatory markers was studied.

**Figure 1:**
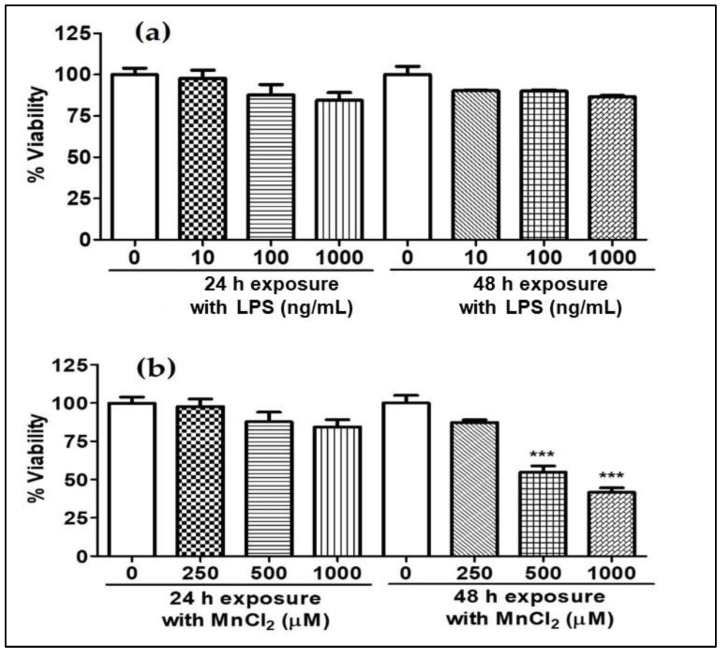
Effect of MnCl_2_ and LPS on viability of N2a cells, (**a**) %viability of N2a cells post-LPS (10, 100 and 1000 ng/mL) exposure for 24 and 48 h, (**b**) %viability of N2a cells post-MnCl_2_ (250, 500 and 1000 µM) exposure for 24 and 48 h. Data represented as mean ± SEM. *** *p* < 0.0001 vs. control group.

N2a cells were treated for 24 h and 48 h with different concentrations of MnCl_2_ (250, 500 and 1000 µM) and LPS (10, 100 and 1000 ng/mL). Our data showed the LPS had no effect on cell viability in any of the tested concentrations (Figure 1a). On the other hand, MnCl_2_ showed a ~50% reduction in cell viability 48 h post-treatment with 500 µM and 1000 µM MnCl_2_ (Figure 1b), indicating the neurocytotoxicological effect of MnCl_2_ on the N2a cell line. 

### 2.2. MnCl_2_ Treatment Induces Gene and Protein Expression of NLRP3 and KHSRP in N2a Cell Line

NLRP3, or NOD-like receptor protein-3, is part of the innate immune system and a component of the NLRP3 inflammasome, which plays a crucial role in neuroinflammation in CNS diseases. KHSRP is a nucleic acid-binding protein that is involved in diverse cellular processes such as transcription, regulation, alternative RNA splicing, cell proliferation, differentiation and metabolism. KHSRP is also found to be involved in neuromuscular disorder, obesity, type-II diabetes and cancer [27].

**Figure 2:**
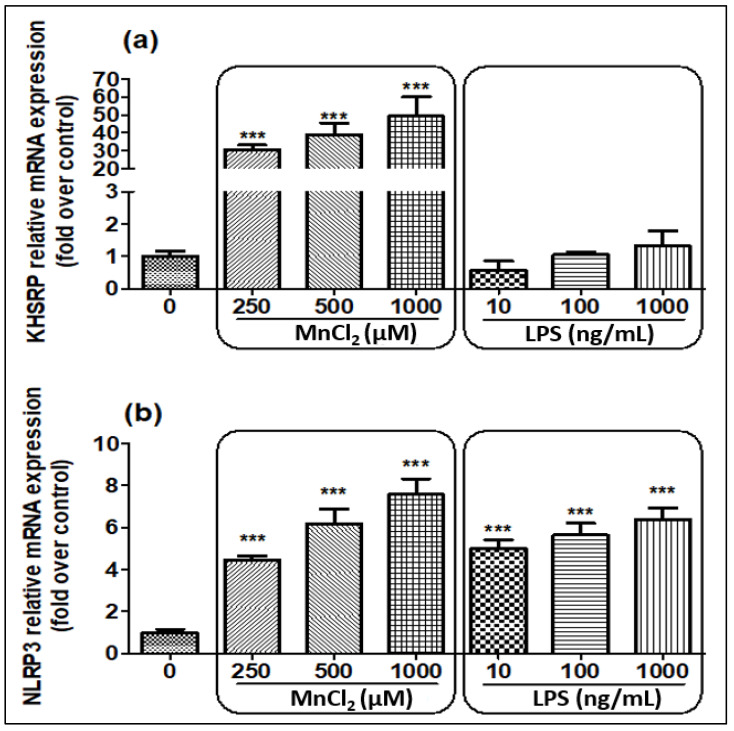
Effect of MnCl_2_ and LPS on mRNA expression of KHSRP and NLRP3 detected by qPCR post-24 h of exposure. (**a**) Relative mRNA expression levels for KHSRP; (**b**) Relative mRNA expression levels for NLRP3. All mRNA values were normalized to the housekeeping mRNA, GAPDH, and expressed as a fold-induction over the control sample (set at a value of 1). Data represented as mean ± SEM. *** *p* < 0.0001 vs. control group.

We evaluated the expression of KHSRP and NLRP3 when N2a cells were treated with MnCl_2_ and LPS (Figure 2a,b) for 24 h. Post-treatment, we detected upregulation of KHSRP expression only with MnCl_2_ treatment, while LPS treatment did not affect the expression of KHSRP expression. On the contrary, NLRP3 expression was upregulated with both MnCl_2_ and LPS at all the tried conditions in a dose-dependent manner.

**Figure 3:**
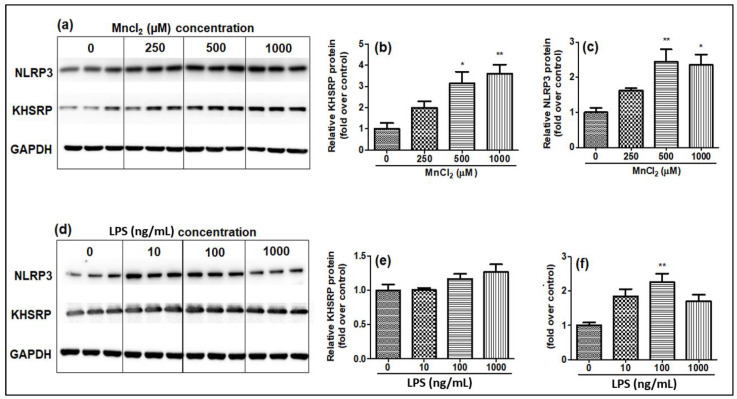
Effect of MnCl_2_ and LPS on protein levels of KHSRP and NLRP3 quantitated by Western blot analysis 24 h post-exposure, relative band intensity quantified (**a**) Western blot analysis for the impact of MnCl_2_ exposure on NLRP3, KHSRP, GAPDH, (**b**) fold change in KHSRP protein levels (relative quantification) post-MnCl_2_ exposure, (**c**) fold change in NLRP3 protein post-MnCl_2_ exposure, (**d**) Western blot analysis for the impact of LPS exposure on NLRP3, KHSRP, GAPDH, (**e**) fold change in KHSRP protein levels (relative quantification) post-LPS exposure, (**f**) fold change in NLRP3 protein post-LPS exposure. Protein intensity values were normalized to the housekeeping protein, GAPDH, and expressed as a fold-induction over the control sample (set at a value of 1). Data represented as mean ± SEM. * *p* < 0.05, ** *p* < 0.001.

Along with mRNA expression, the protein levels of KHSRP and NLRP3 were also studied by Western blot, and the results were observed as similar to the mRNA results, where KHSRP protein expression was induced only by MnCl_2_ treatment (Figure 3a–c) and LPS treatment had no effect on KHSRP protein expression in N2a cell lines (Figure 3e–g). NLRP3 expression was shown to increase after both MnCl_2_ and LPS treatments.

A decrease in RNA and protein quantity and quality was observed post-48 h of treatment; thus, further evaluation was not performed.

Our findings suggested only MnCl_2_ was able to induce KHSRP mRNA expression as well as KHSRP protein positively, whereas LPS did not have any effect on either KHSRP mRNA expression or protein levels. However, both MnCl_2_ and LPS were able to upregulate the NLRP3 mRNA expression and protein levels. The treatment of N2a cells with 500 µM of MnCl_2_ showed consistent results with a good fold increase in comparison to the control group; thus, this dose was selected for further experiments. The LPS was selected as a positive control, since LPS is known for the induction of inflammatory cytokines such as IL-6, IL-1β and NF-κB. The LPS mimics elevated cytokine production, as can be seen in the case of MnCl_2_.

### 2.3. NLRP3 Inhibitor MCC950 Inhibits the Expression of KHSRP

**Figure 4:**
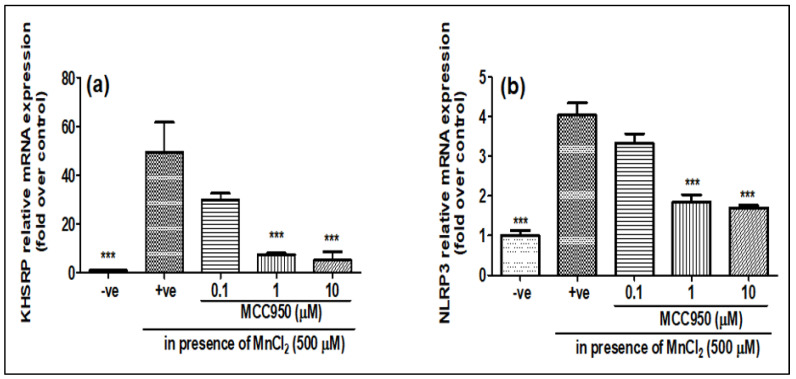
MCC950 inhibited MnCl_2_-induced KHSRP and NLRP3 genes gradually in a dose-dependent manner. N2a cells were pre-treated for 1 h with MCC950 (0.1, 1 and 10 µM) and later exposed to MnCl_2_ (500 µM) for 24 h. (**a**) relative KHSRP mRNA expression; (**b**) relative NLRP3 mRNA expression. All mRNA values were normalized to the housekeeping mRNA, GAPDH, and expressed as a fold-induction over the control sample (set at a value of 1). Data represented as mean ± SEM. *** *p* < 0.0001 vs. control group.

**Figure 5:**
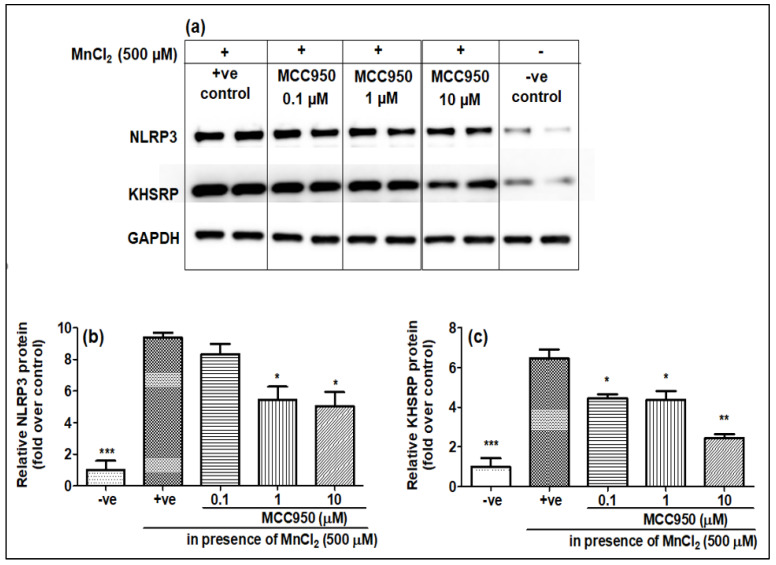
MCC950 inhibited the MnCl_2_-induced KHSRP and NLRP3 protein levels in a dose-dependent manner. N2a cells were pre-treated for 1 h with MCC950 (0.1, 1 and 10 µM) and later exposed to MnCl_2_ (500 µM) for 24 h. (**a**) Western blot images for NLRP3, KHSRP and GAPDH, (**b**) histograms for KHSRP relative protein levels, (**c**) histograms for NLRP3 relative protein levels. All protein values were normalized to the housekeeping protein, GAPDH, and expressed as a fold-induction over the control sample (set at a value of 1). Data represented as mean ± SEM. * *p* < 0.05, ** *p* < 0.001, *** *p* < 0.0001 vs. control group.

As it was seen that MnCl_2_ treatment induces the expression of both NLRP3 and KHSRP while LPS treatment only induces the expression of NLRP3, to see whether the increase in NLRP3 and KHSRP expressions induced by MnCl_2_ are linked, we pre-treated the cells with MCC950 before treatment with 500 μM of MnCl_2_, which is a pharmacological inhibitor of NLRP3. It was found that MCC950 not only inhibited the expression of NLRP3 but also inhibited the expression of KHSRP, both at gene (Figure 4a,b) and protein level (Figure 5a–c). This showed that NLRP3 might have a role in inducing the expression of KHSRP in N2a cells upon MnCl_2_ treatment. However, there have been no previous reports of NLRP3 and KHSRP interaction; the question will be whether KHSRP is upstream or downstream of the NLRP3. As we have seen that the treatment of LPS induces NLRP3, but there is no induction of KHSRP, this indicates that some other pathway could be involved in the induction of KHSRP that needs to be further investigated. Furthermore, an earlier report from Bollmann et al. showed that KHSRP is involved in the regulation of pro-inflammatory gene expression, which is a known effector of NLRP3 [28]. These results, and previous reports, clearly indicate that KHSRP could be targeted by the different signaling effector for NLRP3 for inducing neuroinflammation upon manganese toxicity.

### 2.4. NLRP3 Inhibitor MCC950 Inhibits MnCl_-2_-Induced Gene Expression of Various Pro-Inflammatory Markers and Caspase-1

**Figure 6:**
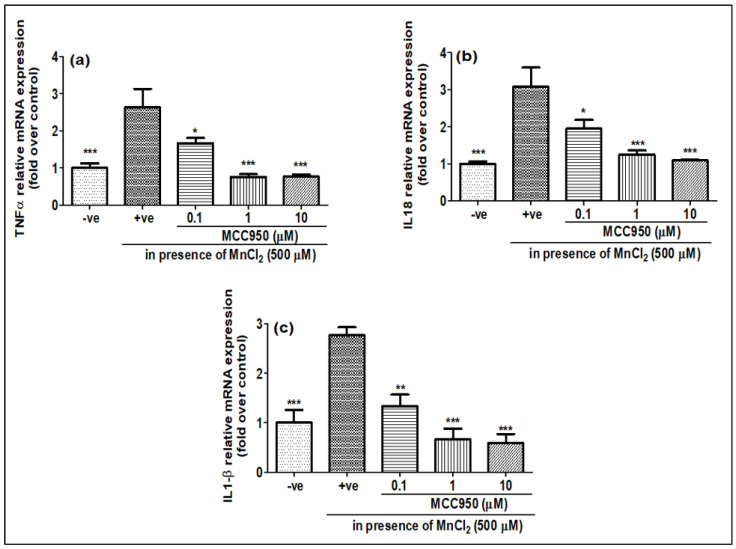
MCC950 was able to inhibit MnCl_2_-induced pro-inflammatory cytokines detected by qPCR. N2a cells were pre-treated for 1 h with MCC950 (0.1, 1 and 10 µM) and later exposed to MnCl_2_ (500 µM) for 24 h. (**a**) relative TNFα gene expression, (**b**) relative IL-18 gene expression, (**c**) relative IL-1β gene expression. All mRNA values were normalized to the housekeeping mRNA, GAPDH, and expressed as a fold-induction over the control sample (set at a value of 1). Data represented as mean ± SEM. * *p* < 0.05, ** *p* < 0.001, *** *p* < 0.0001 vs. control group.

**Figure 7:**
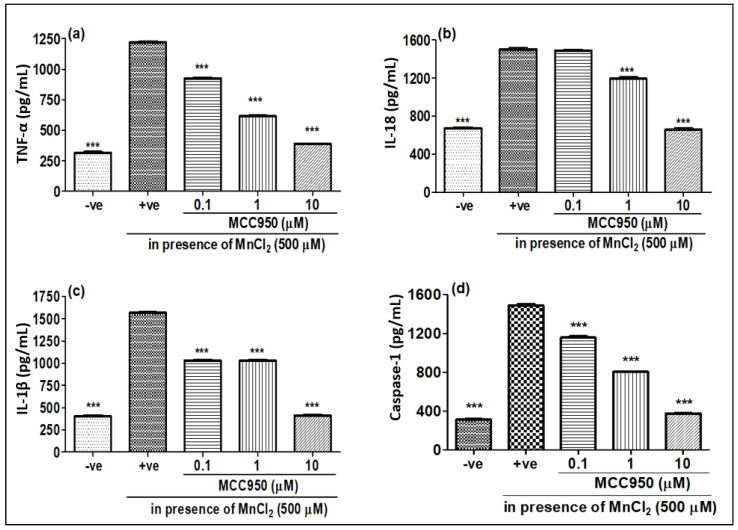
Effect of MCC950 on different cytokines in the presence/absence of MnCl_2_. N2a cells were exposed to MnCl_2_ with/without MCC950 (0.1, 1 and 10 µM) detected by ELISA post 24 h. (**a**) TNFα, (**b**) IL-18, (**c**) IL-1β, (**d**) caspase-1. Data represent the mean ± SD, significance calculated with reference to +ve group, i.e., MnCl_2_ 500 µM. *** *p* < 0.0001 vs. control group.

Mn has been reported to induce neurotoxicity by the release of inflammatory mediators such as pro-inflammatory cytokines and caspase-1. We checked the gene expression as well as release of protein levels of pro-inflammatory cytokines when N2a cells were treated with MnCl_2_ for 24 h. The gene expression of TNF-α, IL-18 and IL-1β showed an increase upon MnCl_2_ treatment (Figure 6a–c). Furthermore, we also observed an increase in the release of pro-inflammatory cytokines such as TNF-α, IL-18 and IL-1β, as well as the protein level of caspase-1 in N2a cells upon treatment with MnCl_2_ (Figure 7a–d). When N2a cells were treated with MCC950, the gene expression of pro-inflammatory markers TNF-α, IL-18 and IL-1β showed a dose-dependent decrease (Figure 6a–c). Similar to the gene expression, the release of these pro-inflammatory markers TNF-α, IL-18 and IL-1β (Figure 7a–c) and the protein level of caspase-1 (Figure 7d) also showed a dose-dependent decrease upon MCC950 pre-treatment. This shows that the increase in the pro-inflammatory markers TNF-α, IL-18 and IL-1β upon MnCl_2_ treatment is NLRP3-dependent.

### 2.5. Evaluation of KHSRP and NLRP3 in Rat Brain after MnCl_2_ Exposure in Presence of MCC950

**Figure 8:**
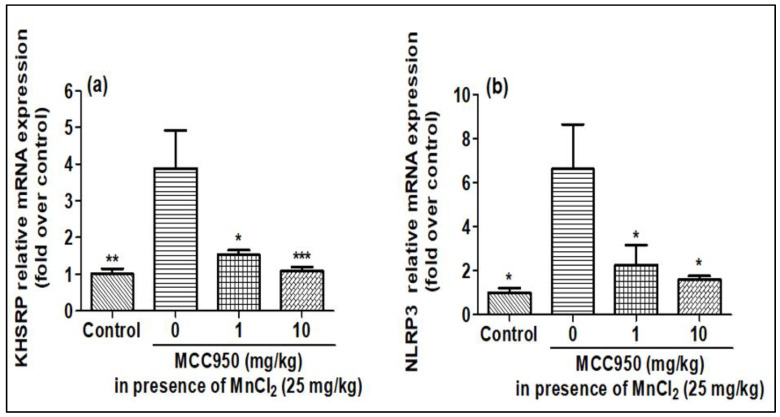
MnCl_2_ was able to overexpress the KHSRP and NLRP3 mRNA expression, which was controlled in a dose-dependent manner by MCC950 in rat brain samples detected by qPCR. (**a**) relative KHSRP mRNA expression, (**b**) relative NLRP3 mRNA expression. All mRNA values were normalized to the housekeeping mRNA, GAPDH, and expressed as a fold-induction over the control sample (set at a value of 1). Data represented as mean ± SEM. * *p* < 0.05, ** *p* < 0.001, *** *p* < 0.0001 vs. control group.

**Figure 9:**
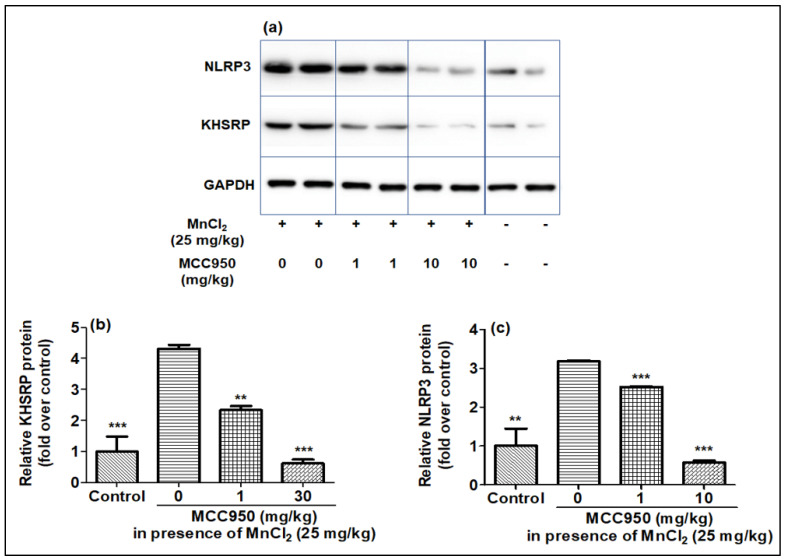
KHSRP, and NLRP3 protein levels were upregulated by MnCl_2_, and further inhibited in a dose-dependent fashion by MCC950 in rat brain samples estimated by Western blot. (**a**–**c**) Western blot images and histograms for KHSRP and NLRP3. Relative protein for KHSRP and NLRP3 protein intensity values calculated post normalization to the housekeeping protein, GAPDH, and expressed as a fold-induction over control sample (set at a value of 1). Data represented as mean ± SEM. ** *p* < 0.001, *** *p* < 0.0001 vs. control group.

The effect of MnCl_2_ on KHSRP and NLRP3 gene expression was explored on SD rats in the presence of MCC950. The qPCR of rat brain samples showed that treatment of MnCl_2_ increased the expression of the NLRP3 and KHSRP genes, which was found to be lessened by MCC950 (Figure 8a,b). Further, the Western blotting of brain samples also showed that expression of NLRP3 and KHSRP increased upon MnCl_2_ treatment and reduced with the treatment of MCC950 (Figure 9a–c). The results of qPCR and Western blot in the rat brain were comparable to our earlier findings as seen in cell lines.

### 2.6. Effect of MnCl_2_ Treatment on Expression of Pro-Inflammatory Markers in Rat Brain in Presence of MCC950

**Figure 10:**
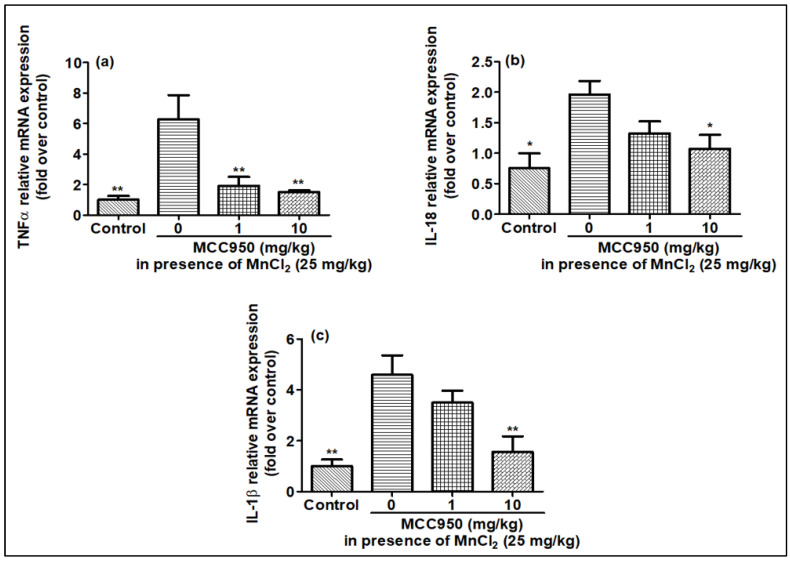
mRNA expression for pro-inflammatory cytokines was elevated by MnCl_2_ exposure and inhibited in a gradual dose-dependent manner by MCC950 in rat brain samples evaluated by qPCR. (**a**) relative TNFα gene expression, (**b**) relative IL-18 gene expression, (**c**) relative IL-1β gene expression. All mRNA values were normalized to the housekeeping mRNA, GAPDH, and expressed as a fold-induction over the control sample (set at a value of 1). Data represented as mean ± SEM. * *p* < 0.05, ** *p* < 0.001 vs. control group.

**Figure 11:**
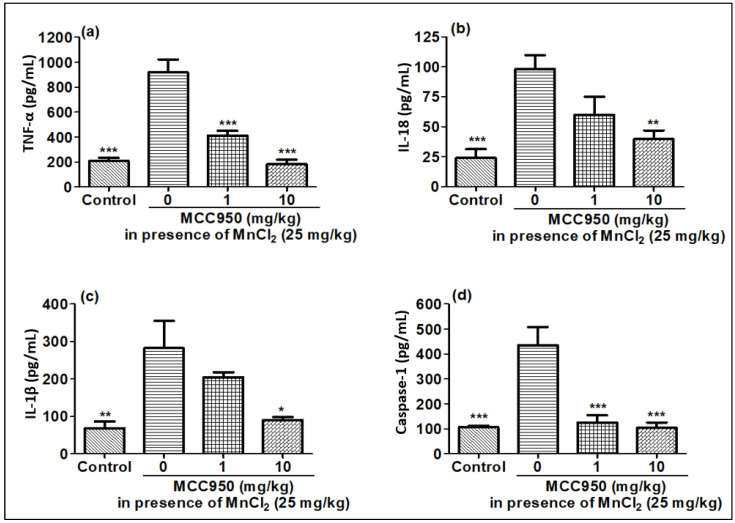
MnCl_2_ exposure elevated pro-inflammatory cytokines, and MCC950 inhibited cytokines in rat brain samples detected by ELISA. (**a**) TNFα, (**b**) IL-18, (**c**) IL-1β, (**d**) caspase-1. Data represented as mean ± SEM. * *p* < 0.05, ** *p* < 0.001, *** *p* < 0.0001 vs. disease control group.

Similar to cell lines, gene expression and release of pro-inflammatory markers were checked in rat brain when exposed to MnCl_2_ in the presence of MCC950. Similar to the cell lines, the expression of the TNF-α IL-18 and IL-1β genes increased in the MnCl_2_-treated rat brain compared to the control rat brain tissue, in harmony with qPCR results (Figure 10a–c). MnCl_2_-treated rat brain tissues also showed an increased release of TNF-α IL-18 and IL-1β cytokines, and also caspase-1 in comparison to control rat brain tissues, in line with qPCR data as well as cell line data (Figure 11a–d). This increase in the production of the pro-inflammatory marker induced by MnCl_2_ treatment was observed to be inhibited in the rats treated with MCC950 (Figure 11a–d). MCC950 also reduced the increased gene expression for these markers in MnCl_2_-treated mice (Figure 10a–c). These results demonstrate that MnCl_2_ neurotoxicity is attributed to neuroinflammation mediated by the release of the pro-inflammatory markers caspase-1, TNF-α IL-18 and IL-1β due to activation of the NLRP3 pathway. KHSRP in its traditional role destabilizes the mRNA of TNF-α IL-18 and IL-1β but in the case of manganese toxicity its role is reversed.

Neurodegeneration is attributed to the malfunctioning of the basic functions of neurons, which leads to neurodegenerative diseases such as Alzheimer’s or Parkinson’s disease. These diseases correspond to the loss of the structure and function of the neurons in the central nervous system. Neurodegeneration has devastating consequences for the mental and physical functioning of the brain. The actual causes of neurodegenerative diseases are unknown; however, it is believed that the major reason is genetic factors or age, and the second major reason for the disease is environmental factors such as neurotoxicant exposure. One of the best-known neurotoxicants is manganese; it is a kind of neurotoxicant whose exposure can produce a form of parkinsonism (also known as manganism) [5]. Manganese is an essential element for human metabolism [2]. However, excessive exposure to manganese could lead to neurodegeneration characterized by striatal dopamine depletion, neuronal loss, marked astrocytosis and extrapyramidal dysfunction [1,5,29]. Neurotoxicity caused by manganese is regulated by multiple factors such as mitochondrial dysfunction, neuroinflammation and misfolding of proteins. Manganese toxicity is characterized by the induction of inflammation. NLRP3 (NOD-, LRR- and pyrin domain-containing protein 3) is a part of the innate immune system [30]. It functions as a pattern-recognition receptor (PRR). It works as an intracellular sensor that detects microbial invasion, cell damage and environmental irritants. Upon activation, it results in the formation of NLRP3 inflammasome, which leads to the release of pro-inflammatory markers. NLRP3 inflammasome could also be activated by manganese in microglial cells [31].

In this study, we demonstrated that exposing mouse neuroblastoma cell line N2a to MnCl_2_ induces the cytotoxicity and upregulation of both the KHSRP and NLRP3 genes and proteins, suggesting the role of both in the toxicity-associated pathway, while on the other hand LPS could induce only NLRP3 expression and not KHSRP, suggesting the absence of a KHSRP role in the pathogen-related pathway. Further, we also demonstrated that this elevated expression of KHSRP and NLRP3 upon MnCl_2_ exposure was inhibited by the treatment of the NLRP3 inhibitor MCC950, indicating the role of NLRP3 in KHSRP expression upon MnCl_2_ treatment. We also demonstrated that inhibiting NLRP3 by MCC950 results in the inhibition of gene expression as well as the release of pro-inflammatory markers such as TNF-α, IL-18 and IL-1β and caspase-1 enzyme, which are elevated upon Mn treatment. In addition to the cellular results, we also assessed the effect of MnCl_2_ in SD rats, where results corresponded with cellular results. Results from the animal study indicated KHSRP and NLRP3 gene expression were elevated with MnCl_2_ exposure and subsided with the treatment of MCC950. Likewise, expression and release of the pro-inflammatory marker gene and protein were also increased with MnCl_2_ exposure and further controlled with MCC950 treatment.

One of the earlier studies reported that direct pulmonary exposure of rats to welding fumes containing manganese not only resulted in pulmonary inflammation and cytotoxicity but high-Mn exposure also resulted in the deposition of Mn brain striata and midbrain [32]. Exposure to Mn, combined with an inflammagen, could enhance the production of iNOS/NO [33,34,35] and PGE2 [36]. It has also been found to stimulate the production of the inflammatory cytokines TNF-α [35,37,38], IL-1β [38] and IL-6 [35] by glial cells. An earlier study by Sarkar et al. showed that manganese toxicity led to the activation of the NLRP3 inflammasome complex in rat microglial cells [31]. This activation led to the induction of the multiprotein NLRP3 inflammasome complex to promote neuroinflammation. Zhao et al showed that Mn exposure facilitated the activation of NLRP3 inflammasome to promote the production of IL-1β and IL-18 in dose- and time-dependent manners in HAPI cells [39]. Data from the current study demonstrated that there was an increase in the expression of the NLRP3 gene and protein expression. Additionally, we demonstrated an increase in the release of caspase-1, TNF-α, IL-18 and IL-1β upon MnCl_2_ treatment.

KHSRP or KSRP is a nucleic acid-binding protein [27]. It has been known to be associated with various cellular processes. It is reported as a multifunctional protein that is involved in neuro-specific alternative splicing [40]. Shi et al. have previously reported an increase in the expression of KHSRP protein in rat striata upon Mn exposure [17]. The one and only study published previously showed that Mn treatment led to the upregulation of KHSRP in PC-12 cells [17]. Manganese-induced KHSRP upregulation along with active caspase-3 led to apoptosis in PC-12 cells. The present study also showed that the Mn treatment up-regulates the expression of KHSRP. However, we have found that this upregulation is dependent on NLRP3 expression.

Mn exposure has been reported to activate the three major MAPKs, i.e., ERK, p38 and JNK, in glial cells, out of which the expression of p38 is stable for a longer time [37,41,42,43,44]. It was found that this activation of MAPK could be the result of the activation of MAPK kinases upstream of MAPK, i.e., MKK-3/6, MKK-1/2 and MKK-4 by Mn, which are responsible for activation of p38, ERK and JNK, respectively [42]. Exposing LPS-primed microglial cells to Mn or Mn exposure in mouse models has also significantly amplified NLRP3, caspase-1 cleavage and IL-1β maturation and enhanced NLRP3 inflammasome activation in microglial cells in vitro and in vivo [31]. Hence, it is possible that Mn treatment could lead to the activation of the MAPK pathway and NLPR3 inflammasome that then activates the KHSRP.

KHSRP is involved in the AU-rich element (ARE)-mediated mRNA decay of pro-inflammatory mediators such as TNF-α [45], interleukin-8 (IL-8) [46] and inducible nitric oxide synthase (iNOS) [21]. The activity of KHSRP is controlled by its phosphorylation by p38 MAPK, ataxia telangiectasia-mutated (ATM) kinase and PI3K-Akt. Resveratrol is a known inhibitor of NLRP3-mediated inflammasome activation [47]. It reduces the p38 MAPK-related inhibitory KHSRP threonine phosphorylation [28]. It further increases the intra-cellular binding of KHSRP to IL-8, iNOS and TNF-α mRNA, thereby enhancing degradation of these mRNAs. These data indicate that the expression of KHSRP and release of these pro-inflammatory markers are reversed to each other. In contrast, in the present investigation we have shown that Mn toxicity leads to an increase in the expression of NLRP3 and thereby increases the release of pro-inflammatory markers and leads to an NLRP3-dependent increase in the expression of KHSRP. However, this increase in KHSRP seems to be helping neuroinflammation caused by NLRP3. The reason might be the changing functionality of KHSRP, which instead of degrading the mRNA of pro-inflammatory markers might be stabilizing these mRNAs. However, this needs to be further investigated.

## 3. Materials and Methods

### 3.1. Cell Culture

The mouse neuroblastoma (N2a) [48] cell line was obtained from ATCC. The cells were cultured in T150 flasks and cultured in Eagle’s Minimum Essential Medium (ATCC) supplemented with 10% fetal bovine serum (FBS, Gibco, MA, USA) and 1% *Penicillin-Streptomycin* (10,000 U/mL, Gibco, Waltham, MA, USA). Cells were incubated in a humidified incubator at 37 °C with 5% CO_2_.

### 3.2. Cell Viability Assay

N2a cells were seeded overnight in clear-bottom black 96-well plates at the density of 10,000 cells/well. The following day, the media were replaced with fresh media before initiating the experiment. Cells were treated with/without LPS (O111:B4, Sigma, Burlington, MA, USA) at the concentration of 10, 100, 1000 ng/mL [49], and with/without MnCl_2_ (Sigma, M1787, MA, USA) at the concentration of 250, 500 and 1000 µM [39]. Cells were treated for 24 and 48 h, and viability was assessed using celltiter glo (Promega, G7570), Wisconsin, USA. The luminescence of plates was read in Envision plate reader from Perkin Elmer. Percent viability was calculated and compared with the control group, i.e., without MnCl_2_/LPS samples vs. MnCl_2_/LPS-treated samples.

### 3.3. Cellular Experiments

N2a cells were seeded in 6-well plates at the density of 0.5 million cells/well and incubated overnight. The following day, media were replaced with fresh media before initiating the experiment. Experiment was divided in two sets; in set 1, cells were treated with/without MnCl_2_ (prepared in sterile PBS) at the concentration of 250, 500 and 1000 µM, and with/without LPS (prepared in sterile PBS) at the concentration of 10, 100, 1000 ng/mL. Cells were incubated for 24 h in a humidified incubator at 37 °C with 5% CO_2_. In set 2, experiment cells were pre-incubated for 1 h with/without MCC950 (prepared in 100% DMSO) at concentrations of 0.1, 1 and 10 µM with the final concentration of DMSO as 1%. The concentration of MCC950 was chosen based on information in the literature [50]. MCC950 post-1 h cells were exposed with/without MnCl_2_ at the concentration of 500 µM (prepared in sterile PBS). Cells were incubated for 24 h in a humidified incubator at 37 °C with 5% CO_2_. Cells were further used for gene expression, protein expression and ELISA experiments.

### 3.4. Animals and Treatment

Adult male Sprague–Dawley (SD) rats (6 weeks old, weighing 180–210 g) were purchased from Vivo Bio Tech Ltd., Telangana, India. All animals were kept under a controlled temperature and environment (23 ± 1 °C, 50 ± 5% humidity) on a 12 h light/dark cycle with free access to water and food. All the experiments were approved by Institutional Animal Ethics Committee (IAEC). Animals were allowed to be acclimatized for 1 week before the initiation of the experiment.

### 3.5. Animal Study Design and Treatment

**Figure 12:**
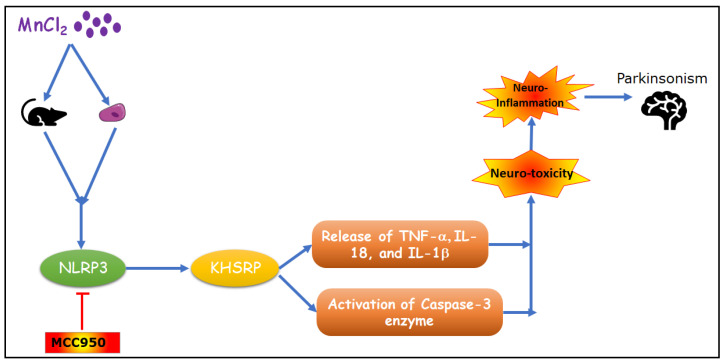
Schematic presentation of MnCl_2_-induced neurotoxicity.

Animals were randomly divided into four groups (12 animals/group): group 1: normal control, group 2: disease control, group 3: MCC950 treatment group 1 mg/kg of body weight and group 4: MCC950 treatment group 10 mg/kg of body weight. Normal control group rats were injected with saline (NaCl, 0.9%), whereas disease control and MCC950 treatment group rats were injected with MnCl_2_ (25 mg/kg body wt, diluted in saline) via intraperitoneal route once, daily for 30 consecutive days, based on the published protocol [17,26]. The schematic presentation of MnCl_2_-induced neurotoxicity is depicted in Figure 12.

Animals received either the vehicle (PBS) or MCC950 (CP-456773 sodium salt, PZ0280, Sigma, MA, USA) prepared in PBS (MCC950-sodium-DataSheet-MedChemExpress, NJ, USA) at 1 mg/kg and 10 mg/kg, po, qd. for 30 days, starting from day 1 to day 30. Animals were sacrificed on the 30th day. The dosages of MCC950 were chosen based on the information in the literature [51]. Body weight for individual animals was recorded twice weekly during the study period. Twenty-four hours after the last injection, animals were sacrificed, and the total brain was collected from the animals and processed for: (1) Western blot analysis (*n* = 2) from each group for estimation of KHSRP and NLRP3 protein levels; (2) quantitative real-time PCR (*n* = 4) to study KHSRP, NLRP3, TNFα, IL-1β and IL-18 gene expression; (3) Cytokine/Chemokine (*n* = 4) for estimation of caspase-1, TNFα, IL-1β and IL-18 by ELISA.

### 3.6. RNA Preparation and Quantitative Real-Time PCR

Cellular experiment: post-incubation, the medium was removed and TRI Reagent^®^ (Sigma, T9424, MA, USA ) solution was added to cells and stored at −80°C before proceeding to RNA isolation. Animal study: brain was collected in DNase- and RNase-free centrifuge tube, the brain was resuspended in TRI Reagent^®^ (Sigma, T9424, MA, USA) solution and tissue homogenization was performed through bead beating by Geno-grinder for 2 min and stored at −80° before proceeding to RNA isolation.

Total RNA was isolated from cells and tissue using RNeasy Mini Kit (Qiagen, 74004, Hilden, Germany). RNA (1 µg) was reverse-transcribed into cDNA using the iScript cDNA Synthesis kit (Bio-rad, 1708891, CA, USA) as per the manufacturer’s protocol. Fifty nanograms of cDNA template was amplified in Applied Biosystem QuantStudio using an iTaq Universal SYBR Green Supermix (Bio-rad, 1725121, CA, USA) under the following conditions: pre-incubation step at 95 °C for 5 min was followed by 45 cycles of denaturation at 95 °C for 10 s, annealing at 60 °C for 10 s and elongation at 72 °C for 10 s. The mRNA expression levels of all samples were normalized to the housekeeping gene (GAPDH) and fold change was calculated using the delta Ct method. The protocol was followed as per the published protocol [52]. RNA sequence for the primers used in qPCR ordered from Origene, details mentioned in the Table 1.

### 3.7. Western Blotting

Cellular experiment: post-incubation, medium was removed and cells were lysed using cell lysis buffer (cell signaling, 9803, Danvers, MA, USA) with phosphatase inhibitor cocktail (Sigma, P2850, MA, USA) and a proteinase inhibitor cocktail (Sigma, P8340, MA, USA). Animal study: brain was collected in RIPA Buffer (cell signaling, 9806, MA, USA) with phosphatase inhibitor cocktail (Sigma, P2850, MA, USA) and a proteinase inhibitor cocktail (Sigma, P8340, MA, USA). The protein concentrations were determined using QuantiPro™ BCA Assay Kit (Sigma, QPBCA, MA, USA). Protein (10 µg) was loaded in each well of 12% SDS-acrylamide gel and run for 1 h at 120 V at room temperature. Separated proteins were transferred to nitrocellulose (NC) filter membranes (Bio-Rad, 1620115, Hercules, CA, USA) at 80 V for 45 min at 4 °C. The membranes were incubated with primary antibodies overnight at 4 °C and probed with HRA-labeled secondary antibodies at room temperature for 2 h. The following antibodies were used: KHSRP Antibody (1:2000, Novus biological, NBP1-18910, Centennial, CO, USA), GAPDH (14C10) Rabbit mAb (1:5000, cell signaling, 2118, MA, USA), Human/Mouse NLRP3/NALP3 Antibody (1:1000, MAB7578, R&D systems, Minneapolis, MN, USA), Anti-rabbit IgG, HRP-linked Antibody (1:7500, cell signaling, 7074, MA, USA). The chemiluminescence signal was detected and captured using Amersham ImageQuant 800, Washington, USA. Western blot protocol was followed as per the published protocol [53].

### 3.8. Measurement of TNFα, IL-1β and IL-18 Estimation

Cellular experiment: post-incubation, the medium was collected from the treated cells for cytokine estimation. Animal study: brain was collected and snap-frozen in liquid nitrogen; later, brain was resuspended in PBS buffer and brain homogenization was performed through bead beating by Geno-grinder for 5 min. Samples were aliquoted and stored at −80 °C to avoid freeze–thaw cycle. Cytokines were measured using a mouse IL-18 (R&D, DY7625 DuoSet ELISA, Minneapolis, MN, USA), mouse IL-1β (R&D, DY401 DuoSet ELISA, Minneapolis, MN, USA), mouse TNFα (R&D, DY410 DuoSet ELISA, Minneapolis, MN, USA) according to manufacturer’s instructions. Absorbance was measured on Clariostar (BMG labtech, Ortenberg, Germany) and levels of cytokines were calculated from standard curves using the GraphPad prism.

### 3.9. Measurement of Caspase-1

Cellular experiment: post-incubation, cells were detached using cell scraper, and collected in PBS buffer; homogenization was performed through bead beating by Geno-grinder for 2 min. Animal study: brain was collected and snap-frozen in liquid nitrogen; later, brain was resuspended in PBS buffer and brain homogenization was performed through bead beating by a geno-grinder for 5 min. Samples were aliquoted and stored at −80 °C, to avoid the freeze–thaw cycle. Caspase-1 was measured using a Mouse caspase-1 ELISA Kit (Novus biological, NBP2-75014, CO, USA) according to manufacturer’s instructions. Absorbance was measured on Clariostar (BMG Labtech, Ortenberg, Germany) and levels of cytokines were calculated from standard curves using the Graph Pad prism.

### 3.10. Statistical Analysis

Data are presented as the means ± S.E.M. A one-way analysis of variance was performed for multiple comparisons, and if there was significant variation between treatment groups, the mean values were compared with the respective control using Dunnett’s test. *p* values less than 0.05 were considered significant.

## 4. Conclusions

To conclude, our results showed for the first-time a correlation between KHSRP and NLRP3. The outcomes of the current study demonstrate that MnCl_2_ upregulates KHSRP mRNA and protein and also neuroinflammatory cytokines. Furthermore, we also demonstrated that MCC950 was able to down-regulate the KHSRP effect in N2a (neuroblastoma) cells as well as in rat brain in vivo. These results hint at the role of KHSRP in neuroinflammation with the aid of NLRP3; thus, further studies are required for a more detailed and better understanding.

## Figures and Tables

**Table 1:** RNA sequence for the primers used in qPCR.

Primer	Sequence
KHSRP Forward: 5′–3′	TCCATCCTGCCTTAGTGGGT
KHSRP Reverse: 5′–3′	TAAGCCTCTGCACCCATCG
GAPDH Forward: 5′–3′	CATCACTGCCACCCAGAAGACTG
GAPDH Reverse: 5′–3′	ATGCCAGTGAGCTTCCCGTTCAG
NLRP3 Forward: 5′–3′	TCACAACTCGCCCAAGGAGGAA
NLRP3 Reverse: 5′–3′	AAGAGACCACGGCAGAAGCTAG
TNFα Forward: 5′–3′	GGTGCCTATGTCTCAGCCTCTT
TNFα Reverse: 5′–3′	GCCATAGAACTGATGAGAGGGAG
IL-18 Forward: 5′–3′	GACAGCCTGTGTTCGAGGATATG
IL-18 Reverse: 5′–3′	TGTTCTTACAGGAGAGGGTAGAC
IL-1β Forward: 5′–3′	TGGACCTTCCAGGATGAGGACA
IL-1β Reverse: 5′–3′	GTTCATCTCGGAGCCTGTAGTG

## Data Availability

All the data generated and analyzed during this research project are included in this article. Correspondence and request for material should be addressed to corresponding author Sunil S. More.
